# Clinical effectiveness of scleral taping combined with perfluoropropane for complex retinal detachment

**DOI:** 10.3389/fmed.2025.1722973

**Published:** 2025-11-18

**Authors:** Xin Wang, Chen Li, Linlin Liu, Linying Xie, Yiping Jiang

**Affiliations:** 1 The First Clinical Medical College of Gannan Medical University, Ganzhou, China; 2 The First Affiliated Hospital of Soochow University, Suzhou, China; 3 First Affiliated Hospital of Gannan Medical University, Ganzhou, China

**Keywords:** scleral buckling, C3F8 gas, complex retinal detachment, clinical effectiveness, visual acuity outcomes, intraocular pressure, ophthalmic surgery

## Abstract

**Purpose:**

This study evaluated the effectiveness of scleral taping combined with C3F8 gas filling for the management of complex retinal detachment (CRD) and to objectively assessed its clinical value.

**Methods:**

Forty consecutive patients (40 eyes) with CRD who underwent surgical treatment were included. Patients were divided into two groups: the study group underwent scleral buckling (SB) combined with C3F8 gas filling, and the control group received scleral buckling alone. Each group comprised 20 patients (20 eyes). After anaesthesia, traction sutures were placed at the rectus muscle in all patients. Retinal breaks and degenerative areas were examined and treated with cryotherapy. A silicone sponge was positioned on the scleral surface, and the sutures were tightened to produce an indenting effect. Additionally, patients in the study group received an intravitreal injection of C3F8 gas.

**Results:**

Both uncorrected visual acuity (UCVA) and best-corrected visual acuity (BCVA) improved in the study and control groups on the first post-operative day. However, the improvement was statistically significant only in the control group. Visual acuity in both groups improved significantly at 1 week, 1 month, and 3 months post-operatively compared with baseline and the first post-operative day. Intraocular pressure (IOP) in the study group was significantly elevated at 1 week, 1 month, and 3 months post-operatively compared with baseline. The retinal reattachment rate was 95% in the study group, significantly higher than 70% in the control group. The recurrence rate was 5% in the study group. No serious complications, including retinal incarceration, haemorrhage, anterior segment ischemia, or infection, were observed in either group.

**Conclusion:**

Scleral buckling combined with C3F8 gas filling is a safe and effective treatment for CRD. It significantly improves post-operative visual acuity, enhances retinal reattachment, reduced the recurrence rate with minimal complications.

## Introduction

Complex retinal detachment (CRD)—encompassing rhegmatogenous retinal detachment (RRD), tractional detachment secondary to proliferative diabetic retinopathy (PDR), and detachment following open-globe trauma—is a major cause of irreversible blindness. Without prompt intervention it may lead to phthisis bulbi and permanent vision loss (1, 2). The therapeutic goals are to seal retinal breaks, relieve vitreoretinal traction, achieve stable reattachment, and maximise visual recovery (3–5). Surgery remains the cornerstone of CRD management; timely intervention restores outer retinal anatomy and significantly improves visual outcomes (6, 7). Current surgical options comprise pars plana vitrectomy (PPV), scleral buckling (SB), and pneumatic retinopexy (PR) (8, 9).

SB creates an inward scleral indentation by suturing a silicone band or sponge to the episclera, thereby approximating the neurosensory retina to the retinal pigment epithelium. Being an extra-ocular procedure, SB avoids intra-ocular manipulation, carries a low complication profile, requires minimal specialised equipment, and is cost-effective (10–12). SB is highly effective for uncomplicated detachments with a limited number of small breaks (13, 14). Anatomical success with a single SB procedure for CRD has been reported at about 80% (15). Nevertheless, SB alone is insufficient for complex RRD, which usually requires vitrectomy (16, 17). Studies indicate that vitrectomy achieves a retinal reattachment rate of 89% in the treatment of CRD (18). Vitrectomy necessitates prolonged face-down positioning and a second operation for silicone-oil removal about 6 months later. Even after oil extraction, recurrent detachment may occur, imposing additional psychological and economic burdens and exposing patients to higher complication rates (19, 20).

Intravitreal gas injection, introduced by Hilton in the mid-1980s (21, 22), yields an 85.2% single-operation success rate for uncomplicated RRD (23). The gas bubble tamponades retinal breaks by surface tension, preventing fluid flux into the sub-retinal space and facilitating closure (24–26). No consensus exists regarding gas type or concentration. Low-concentration C3F8 is rapidly absorbed and may provide insufficient tamponade for complex detachments with multiple or large breaks (27–29). In contrast, pure C3F8 is expansile and provides prolonged tamponade owing to its extended intra-ocular half-life (30). Combining SB with pure C3F8 avoids the adverse events associated with silicone-oil tamponade after vitrectomy. Most studies on SB combined with pure C3F8 have primarily focused on patients with uncomplicated retinal detachment, achieving a retinal reattachment rate of 77.6% (31). Research on SB combined with pure C3F8 for the treatment of CRD remains relatively scarce at present. For patients with CRD, intravitreal injection of an expansile gas combined with SB may enhance reattachment rates and maximise visual benefit through sustained internal tamponade. This study evaluates the anatomical and functional outcomes of SB combined with pure C3F8 injection for CRD, aiming to inform surgical decision-making.

## Methods

### Study subjects

This retrospective cohort study employed propensity score matching (PSM) to simulate randomisation. During the analysis phase, multivariable regression analysis was utilised to control for confounding factors including age, degree of proliferative vitreoretinopathy (PVR), number and size of retinal tears, and extent of retinal detachment. Included 40 patients (40 eyes) with CRD who underwent primary surgery at the First Affiliated Hospital of Gannan Medical University between January 2024 and December 2024. Assign patients to either the study group, which underwent SB combined with C3F8 gas filling, or the control group, which underwent SB alone. Each group comprised 20 patients (20 eyes).

Proliferative vitreoretinopathy (PVR) severity was graded according to the classification system established by the American Retinal Society in 1983. Eyes were eligible if they met all of the following: (1) PVR grade B–C; (2) ≥2 retinal breaks located at different meridians and involving more than one quadrant; (3) posterior pole breaks located 2–5 mm anterior to the equator; (4) giant retinal breaks spanning 70–130° circumference; and (5) all breaks distributed between the 8 and 4 o'clock meridians.

Exclusion criteria were: (1) contraindications to surgery; (2) history of prior vitrectomy; (3) significant media opacity precluding fundus examination; (4) failure to attend timely post-operative follow-up; and (5) pre-operative high intraocular pressure (IOP), glaucoma, diabetes, high myopia, or ocular trauma.

All patients were evaluated pre-operatively and at 1 day, 1 week, 1 month, and 3 months post-operatively. Data collected included uncorrected visual acuity (UCVA), best-corrected visual acuity (BCVA), and IOP, as well as enhanced OCT, fundus photography, and indirect ophthalmoscopy findings.

This study adhered to the principles of the Declaration of Helsinki and received approval from the Ethics Committee of the First Affiliated Hospital of Gannan Medical University. Ethical Approval No. : LLSC-2025-069.

### Surgical methods and treatment plan

After comprehensive counselling, written informed consent was obtained from each patient. All operations were performed by the same surgeon. The patient is positioned supine, followed by retrobulbar anaesthesia. The eyelids are retracted using an eyelid speculum. The bulbar conjunctiva is dissected circumferentially 2 mm beyond the scleral-corneal junction to fully expose the scleral area. Retraction sutures are placed on the superior, inferior, medial, and lateral rectus muscles of the operative eye. Under binocular indirect ophthalmoscopy the fundus was systematically inspected; all breaks and clinically significant areas of degeneration were localised and marked on the sclera. Trans-scleral cryotherapy was applied around each break and degenerate retina until a uniform white frost (moderate-grade freeze) was observed. Perform scleral fixation around the tear. Perform scleral puncture drainage in the retinal detachment area, avoiding the retinal tear site. A silicone sponge explant was trimmed to size, positioned parallel to the limbus directly over the break, and secured with pre-placed 5-0 polyester sutures tightened to produce an inward scleral indentation. Following these steps, the experimental group underwent anterior chamber paracentesis at the superior temporal limbus to aspirate an appropriate volume of aqueous humour. Pure C3F8 inert gas (0.3–0.5 ml) was aspirated and injected intraocularly at the 10 o'clock position, 3.5 mm from the limbus, perpendicular to the ocular wall, slowly advancing the gas towards the ocular centre. The bulbar conjunctiva was sutured intermittently and irrigated with a gentamicin and dexamethasone mixture. Tobramycin-dexamethasone ophthalmic ointment was applied to the operated eye, and the patients were instructed to maintain a face-down position for 1–2 weeks and to avoid strenuous activity.

### Statistical methods

Data were analysed using SPSS 27.0. After testing for normality, the mean and standard deviation of continuous variables were calculated. Quantitative variables (UCVA, BCVA, and IOP) were expressed as mean ± standard deviation (*x* ± *s*). For repeated measures (UCVA, BCVA, and IOP), repeated-measures analysis of variance (ANOVA) was performed. Independent-samples t-test were used to compare intergroup differences in UCVA, BCVA, and IOP between the study and control groups, while Fisher's exact test was used to compare intergroup differences in retinal reattachment rate. A *P* < 0.05 was considered statistically significant. Effect sizes are represented by *Cohen's d, partial* η^2^*, and Odds Ratio (OR)*. *Cohen's d* ranges from 0 to 1, with values below 0.5 indicating a small effect, 0.5–0.8 denoting a medium effect, and above 0.8 signifying a large effect. The *partial* η^2^ ranges from 0 to 1, with values between 0.01 and 0.06 indicating a small effect, between 0.06 and 0.14 representing a medium effect, and greater than 0.14 signifying a large effect. *OR* = 1 indicates no association, *OR* < 1 denotes a negative association, *OR* > 1 signifies a positive association, and a higher *OR* value indicates a stronger association.

## Results

### General information

Forty patients with CRD were included in this study, comprising 22 males (55%) and 18 females (45%). Ages ranged from 44 to 84 years, with a mean of 57.2 ± 9.26 years. Twenty-nine patients (72.5%) had two lacunae, and 11 patients (27.5%) had three lacunae. PVR grade B was identified in 31 patients (77.5%)-−15 in the study group and 16 in the control group—while grade C was identified in nine patients (22.5%)—five in the study group and four in the control group. Regarding the extent of detachment, 22 patients (55%) had two quadrants involved, 17 patients (42.5%) had three quadrants involved, and one patient (2.5%) had four quadrants involved.

### Comparative analysis of UCVA, BCVA and IOP before and after surgery

#### Comparative analysis of UCVA before and after surgery

(1) Study group: pre-operative, and 1 day, 1 week, 1 month, and 3 months post-operative mean UCVA values were (1.88 ± 0.37) logMAR, (1.55 ± 0.5) logMAR, (1.26 ± 0.37) logMAR, (0.97 ± 0.34) logMAR, and (0.6 ± 0.26) logMAR, respectively. At 1 day post-operatively, mean UCVA improved compared with pre-operative values, but the difference was not statistically significant (*P* > 0.05). UCVA at 1 week, 1 month, and 3 months was significantly better than at pre-operative and 1-day (*P* < 0.01). At 3 months, UCVA was also significantly better than at 1 week and 1 month (*P* < 0.01, η^2^ = 0.896).
(2) Control group: pre-operative, and 1 day, 1 week, 1 month, and 3 months post-operative mean UCVA values were (1.92 ± 0.34) logMAR, (1.57 ± 0.46) logMAR, (1.37 ± 0.45) logMAR, (1.0 ± 0.23) logMAR, and (0.64 ± 0.18) logMAR, respectively. At 1 day post-operatively, mean UCVA improved compared with pre-operative values, with the difference statistically significant (*P* < 0.05). UCVA at 1 month was significantly better than pre-operative, 1-day, and 1-week values (*P* < 0.05). At 3 months, UCVA was significantly better than pre-operative, 1-day, 1-week, and 1-month values (*P* < 0.01, η^2^ = 0.924).

#### Comparative analysis of BCVA before and after surgery

**Figure 1:**
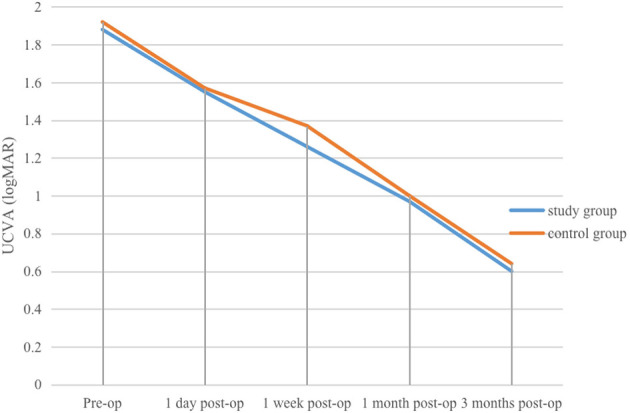
Changes in UCVA at various pre-operative and post-operative time points in the study and control groups.

**Figure 2:**
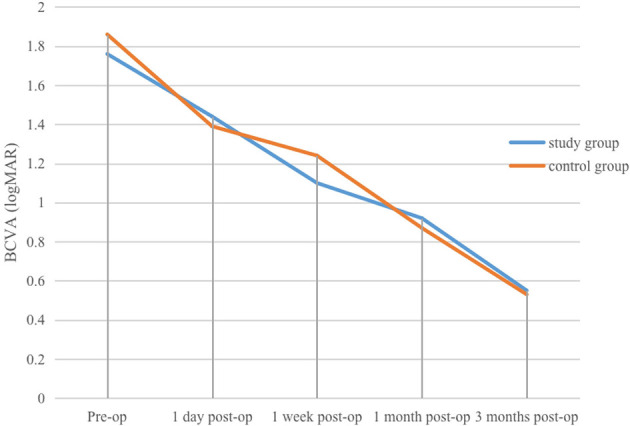
Changes in BCVA at various pre-operative and post-operative time points in the study and control groups.

(1) Study group: pre-operative, and 1 day, 1 week, 1 month, and 3 months post-operative mean BCVA values were (1.76 ± 0.54) logMAR, (1.44 ± 0.59) logMAR, (1.1 ± 0.37) logMAR, (0.92 ± 0.35) logMAR, and (0.55 ± 0.27) logMAR, respectively. At 1 day post-operatively, mean BCVA improved compared with pre-operative values, but the difference was not statistically significant (*P* > 0.05). At 1 week, 1 month, and 3 months, BCVA was significantly better than at pre-operative and 1 day (*P* < 0.01). At 3 months, BCVA was also significantly better than at 1 week and 1 month (*P* < 0.01, η^2^ = 0.825).
(2) Control group: pre-operative, and 1 day, 1 week, 1 month, and 3 months post-operative mean BCVA values were (1.86 ± 0.38) logMAR, (1.39 ± 0.52) logMAR, (1.24 ± 0.49) logMAR, (0.87 ± 0.21) logMAR, and (0.53 ± 0.16) logMAR, respectively. At 1 day post-operatively, mean BCVA improved compared with pre-operative values, with the difference statistically significant (*P* < 0.05). At 1 month, mean BCVA was significantly better than at pre-operative, 1-day, and 1-week *(P* < 0.05). At 3 months, mean BCVA was significantly better than at pre-operative, 1-day, 1-week, and 1-month (*P* < 0.01, η^2^ = 0.908). Within-group differences in UCVA and BCVA at different time points in the control group are shown in Table 1. Within-group differences in UCVA and BCVA in the study group are presented in Table 2, and changes in UCVA and BCVA in both groups are shown in Figures 1, 2.

**Table 1:** Comparison of within-group differences in UCVA and BCVA at different times between pre-operative and post-operative periods in the control group ($\bar{x}\pm s$).

**Time**	**UCVA**	**BCVA**
Pre-op	1.92 ± 0.34a	1.86 ± 0.38a
1 day post-op	1.57 ± 0.46b	1.39 ± 0.52b
1 week post-op	1.37 ± 0.45b	1.24 ± 0.49b
1 month post-op	1.0 ± 0.23bc	0.87 ± 0.21bc
3 months post-op	0.64 ± 0.18d	0.53 ± 0.16d
*F*	48.944	39.657
*P*	0.000	0.000
*Partial η^2^*	0.924	0.908

Identical letters (e.g., a and a) indicate no statistically significant difference (P > 0.05). Different letters (e.g., a and b) indicate statistically significant differences (P < 0.05).

**Table 2:** Comparison of within-group differences in UCVA and BCVA at different times between pre-operative and postoperative periods in the study group ($\bar{x}\;\pm \;s$).

**Time**	**UCVA**	**BCVA**
Pre-op	1.88 ± 0.37a	1.76 ± 0.54a
1 day post-op	1.55 ± 0.5a	1.44 ± 0.59a
1 week post-op	1.26 ± 0.37b	1.1 ± 0.37b
1 month post-op	0.97 ± 0.34bc	0.92 ± 0.35bc
3 months post-op	0.6 ± 0.26d	0.55 ± 0.27d
*F*	36.597	18.92
*P*	0.000	0.000
*Partial η^2^*	0.896	0.825

Identical letters (e.g., a and a) indicate no statistically significant difference (P > 0.05). Different letters (e.g., a and b) indicate statistically significant differences (P < 0.05).

#### Comparative analysis of IOP before and after surgery

(1) Study group: before surgery, four patients (20%) had IOP < 10 mmHg, 16 patients (80%) had IOP 10–21 mmHg, and the mean IOP was (12.18 ± 4.33) mmHg. On the first post-operative day, one patient (5%) had IOP < 10 mmHg, 15 patients (75%) had IOP 10–21 mmHg, and four patients (20%) had IOP >21 mmHg, with a mean IOP of (16.8 ± 6.67) mmHg. At 1 week post-operatively, one patient (5%) had IOP < 10 mmHg, 19 patients (95%) had IOP 10–21 mmHg, and the mean IOP was (15.48 ± 3.35) mmHg. At 1 and 3 months after surgery, IOP in all patients was within the normal range, with mean values of (14.55 ± 1.99) mmHg and (14.3 ± 2.45) mmHg, respectively. Mean IOP at 1 day, 1 week, 1 month, and 3 months was slightly higher than the pre-operative value, with the increase most marked on the first post-operative day, followed by a gradual decline and stabilisation. The difference between IOP on day 1 and pre-operative IOP was not statistically significant (*P* > 0.05), whereas differences at 1 week, 1 month, and 3 months compared with pre-operative values were statistically significant (*P* < 0.05).
(2) Control group: before surgery, two patients (10%) had IOP < 10 mmHg, 18 patients (90%) had IOP 10–21 mmHg, and the mean IOP was (13.5 ± 3.85) mmHg. On the first post-operative day, one patient (5%) had IOP < 10 mmHg, 16 patients (80%) had IOP 10–21 mmHg, and three patients (15%) had IOP >21 mmHg, with a mean IOP of (16.6 ± 5.57) mmHg. At 1 week, 1 month, and 3 months after surgery, all patients' IOP values were within the normal range, with mean values of (15.35 ± 3.00) mmHg, (15.25 ± 2.43) mmHg, and (14.85 ± 2.43) mmHg, respectively. Mean IOP at each post-operative time point was slightly higher than the pre-operative value, with the rise most marked on the first post-operative day, after which IOP gradually decreased and stabilised. The difference in IOP between pre-operative and post-operative time points was not statistically significant (*P* > 0.05).

**Figure 3:**
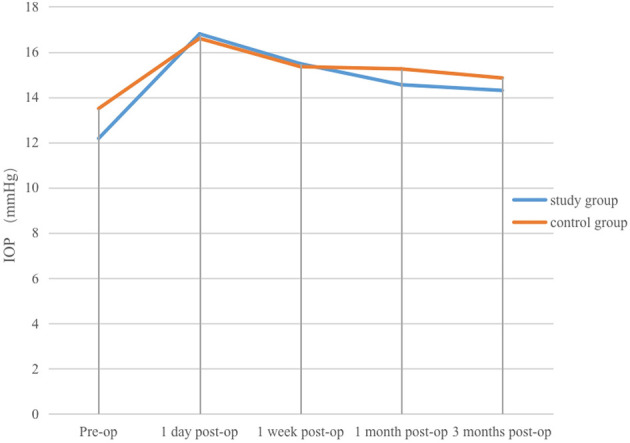
Changes in IOP at various pre-operative and post-operative time points in the study and control groups.

Comparison of intragroup differences in IOP between pre-operative and post-operative time points in the study and control groups is presented in Table 3, and changes in IOP in both groups are shown in Figure 3. Independent-samples *t*-test results for UCVA, BCVA, IOP, and other indices demonstrated no statistically significant intergroup differences at any post-operative time point (*P* > 0.05). Comparative analysis of intergroup differences in each index is presented in Table 4.

**Table 3:** Comparison of intragroup differences in IOP at different times between pre-operative and post-operative periods in the study and control groups ($\bar{x}\;\pm \;s$).

**Time**	**IOP**	
	**Study group**	**Control group**
Pre-op	12.18 ± 4.33a	13.5 ± 3.85a
1 day post-op	16.8 ± 6.67a	16.6 ± 5.57a
1 week post-op	15.48 ± 3.35b	15.35 ± 3a
1 month post-op	14.55 ± 1.99bc	15.25 ± 2.43a
3 months post-op	14.3 ± 2.45d	14.85 ± 2.43a
*F*	3.944	1.51
*P*	0.021	0.246

Identical letters (e.g., a and a) indicate no statistically significant difference (P > 0.05). Different letters (e.g., a and b) indicate statistically significant differences (P < 0.05).

**Table 4:** Comparative analysis of intergroup differences between the two groups on various indicators ($\bar{x}\pm s$).

**Time**	**Study group (*n* = 20)**	**Control group (*n* = 20)**	***t***	***P***	**Cohen's *d***
**UCVA**					
Pre-op	1.88 ± 0.37	1.92 ± 0.34	−0.347	0.731	0.113
1 day post-op	1.55 ± 0.5	1.57 ± 0.46	−0.105	0.917	0.034
1 week post-op	1.26 ± 0.37	1.37 ± 0.45	−0.824	0.415	0.267
1 month post-op	0.97 ± 0.34	1 ± 0.23	−0.299	0.767	0.097
3 months post-op	0.6 ± 0.26	0.64 ± 0.18	−0.631	0.532	0.205
**BCVA**					
Pre-op	1.76 ± 0.54	1.86 ± 0.38	−0.673	0.505	0.218
1 day post-op	1.44 ± 0.59	1.39 ± 0.52	0.295	0.769	0.096
1 week post-op	1.1 ± 0.37	1.24 ± 0.49	−1.068	0.292	0.347
1 month post-op	0.92 ± 0.35	0.87 ± 0.21	0.596	0.555	0.193
3 months post-op	0.55 ± 0.27	0.53 ± 0.16	0.288	0.775	0.093
**IOP (mmHg)**					
Pre-op	12.18 ± 4.33	13.5 ± 3.85	−1.023	0.313	0.332
1 day post-op	16.8 ± 6.67	16.6 ± 5.57	0.103	0.919	0.033
1 week post-op	15.48 ± 3.35	15.35 ± 3	0.124	0.902	0.04
1 month post-op	14.55 ± 1.99	15.25 ± 2.43	−0.999	0.324	0.324
3 months post-op	14.3 ± 2.45	14.85 ± 2.43	−0.712	0.481	0.231

### Post-operative retinal reset rate

On the 1st day, 1 week, 1 month, and 3 months after surgery, fundus examination showed that the fissures were located on the pressurised ridge, were well-closed, and that the retinas were reattached. In the study group, 19 patients (95%) achieved reattachment, whereas in the control group, 70% of patients achieved retinal reattachment. Post-operative recurrence of retinal detachment occurred in one patient (5%) in the study group and in six patients (30%) in the control group. The difference in retinal reset rate between the two groups was statistically significant (*P* < 0.05, *OR* = 8.14).

**Figure 4:**
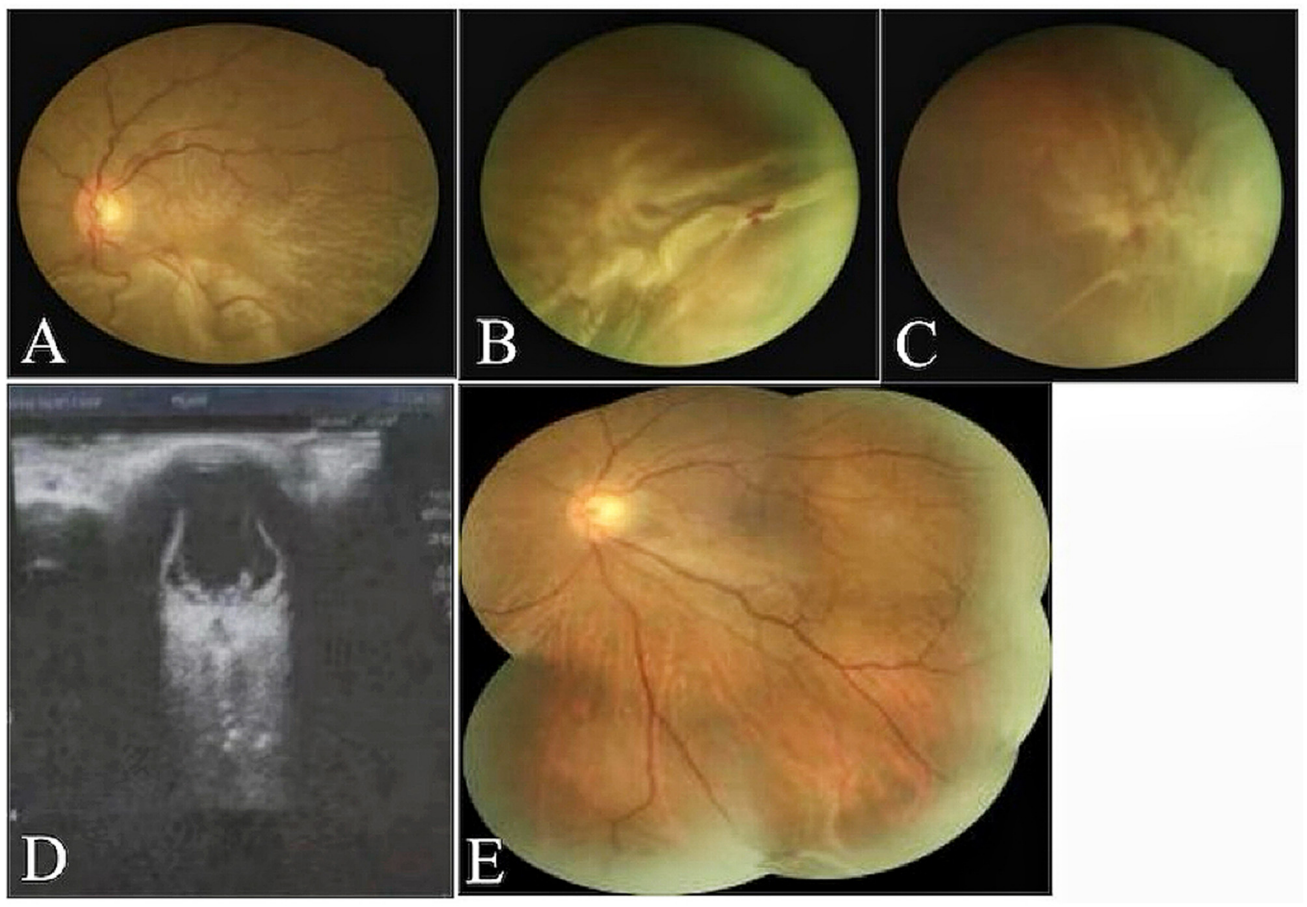
**(A–D)** Fundus photography and ocular ultrasound demonstrated pre-operative total retinal detachment and proliferative retinal changes in the patient. **(E)** One month post-operative complete retinal reattachment in patients undergoing combined SB and pure C3F8 gas filling.

**Figure 5:**
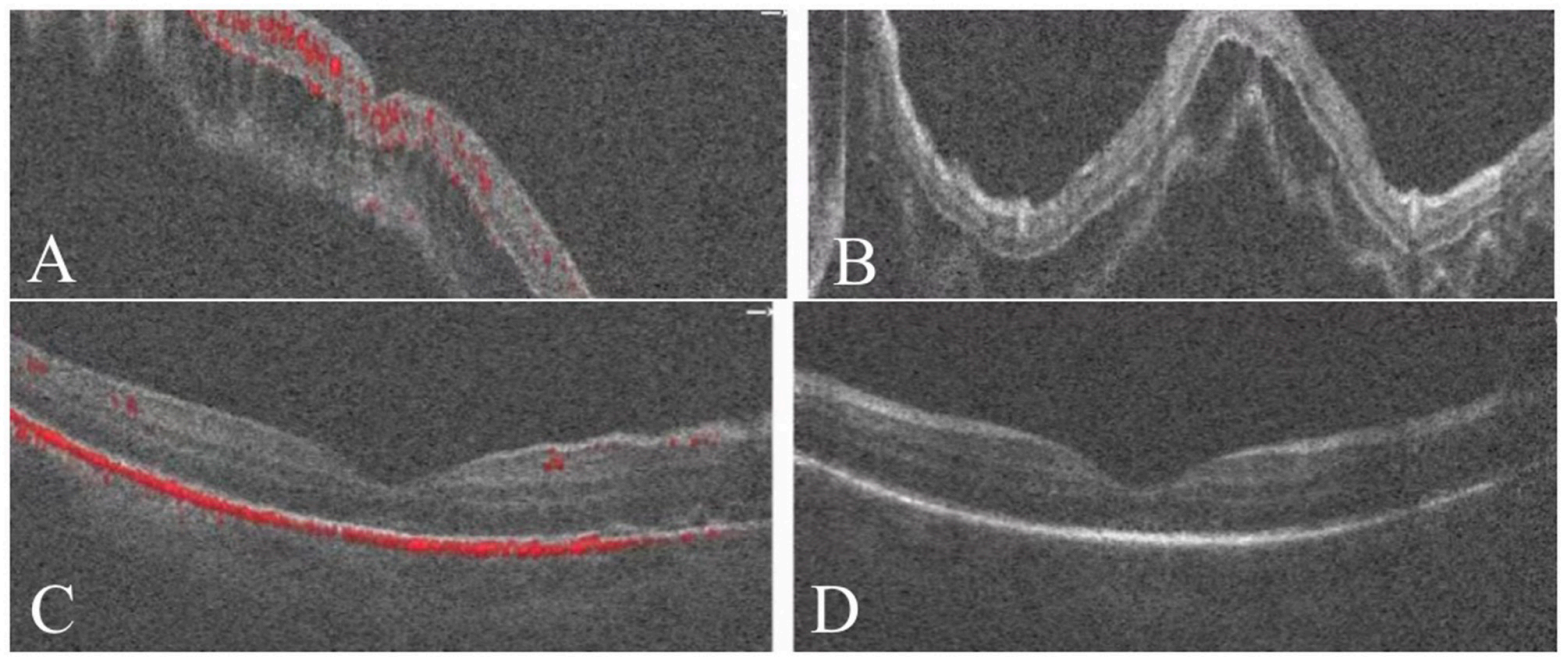
**(A, B)** OCT revealed pre-operative separation of the retinal neuroepithelium from the pigment epithelium, with a subretinal fluid-filled area. **(C, D)** One month post-operatively, retinal reattachment was achieved with absorption of the subretinal fluid and no significant fluid accumulation observed.

Comparison of fundus photography and OCT findings before and after SB combined with C3F8 surgery is shown in Figures 4, 5.

## Discussion

CRD presents a major challenge in ophthalmology and has a profoundly negative impact on patients' vision and quality of life. Vision loss not only limits daily activities but also delivers a heavy psychological burden, increasing anxiety and depression, while simultaneously imposing an economic strain on both families and society (32, 33). CRD is frequently associated with high-risk factors such as giant lacunae, multiple lacunae, and severe PVR. Among these, PVR is particularly important, as it arises from intraocular inflammation and fibroproliferative processes that drive retinal fibrosis (34, 35). Identifying safer and more effective treatment strategies, therefore, remains a key priority for both ophthalmologists and researchers.

Currently, there is no consensus on the optimal surgical modality for patients with CRD. Vitrectomy is an important treatment option, but it increases the risk of post-operative complications, whereas SB is associated with a comparatively lower complication rate and faster visual recovery (36, 37). C3F8 gas has favourable expansion properties and, when injected into the eye, can occupy space to exert a continuous tamponade effect on the retina, thereby facilitating reattachment (38). It also has good biocompatibility and does not provoke significant inflammatory or immune rejection responses within the eye (39). The expansion and absorption dynamics of C3F8 provide effective support for retinal repositioning over a sustained period. With appropriate post-operative positioning, the tamponade effect of C3F8 further accelerates retinal reattachment. In this study, all patients who underwent the combined surgical approach were instructed to maintain the foramen magnum position for 1–2 weeks after surgery, and to avoid strenuous activity, to ensure effective gas compression and stable retinal apposition. In contrast, other intraocular fillers may require a longer healing and recovery process. For example, patients who undergo silicone oil filling must remain in a specific position for a prolonged period, and subsequent silicone oil removal surgery can further extend the recovery phase and increase patient discomfort (40, 41). The volume of C3F8 gas injected is a critical determinant of surgical outcome. If the volume is insufficient, it cannot provide adequate tamponade on the retina, which may result in incomplete reattachment. Conversely, excessive volume may cause a marked rise in IOP, potentially damaging the optic nerve and retina and worsening visual impairment (42) Research indicates that axial length correlates positively with vitreous cavity volume. A longer axial length corresponds to a larger vitreous cavity volume, thus requiring a greater volume of gas to achieve equivalent filling (43). In clinical practise, the dosage of C3F8 gas injection should be adjusted according to the patient's axial length. For patients with shorter axial lengths (< 24 mm), 0.3–0.4 ml of C3F8 gas may be administered. For patients with longer axial lengths (≥24 mm), 0.4–0.5 ml of C3F8 gas may be administered. In this study, all patients received 0.3–0.5 ml of gas, tailored to their ocular condition, and no intraoperative spikes in IOP were observed.

The results of this study demonstrated that the study group achieved superior outcomes in promoting retinal reattachment. Post-operative funduscopic examination showed that fissures were located on the pressurised ridge, were well-closed, and the retina was flat in 14 patients in the control group, giving a reattachment rate of 70%. Six patients experienced recurrent retinal detachment after surgery, with a recurrence rate of 30%. In the study group, 19 patients achieved post-operative retinal reattachment, corresponding to a reattachment rate of 95%, which exceeded the retinal reattachment rate observed in vitrectomy procedures (18), only one patient experienced recurrent retinal detachment, giving a recurrence rate of 5%. This difference may be attributable to the effect of pure C3F8 gas injected into the vitreous cavity, which exerted an effective tamponade on the retina, compensating for the insufficient local pressure of simple SB and thereby stabilising larger retinal areas to reduce recurrence.

Among the seven patients with recurrence, two had PVR grade B and five had grade C. The recurrence rates in patients with PVR grade B and C were 6.4 and 55.6%, respectively. Among those with two retinal holes, three patients relapsed (recurrence rate 10.3%), whereas among those with three retinal holes, four patients relapsed (recurrence rate 36.3%). For detachments spanning two quadrants, recurrence occurred in two patients (9.1%); for three quadrants, four patients relapsed (23.5%); and for four quadrants, one patient relapsed (100%). These findings demonstrate that more severe PVR, a greater number of retinal breaks, and larger detachment extent are associated with higher recurrence rates and reduced surgical success (44).

In terms of visual acuity, no statistically significant difference was found between the two groups, yet a marked disparity in retinal reattachment rates was observed. This may indicate that the advantage of combined surgery lies in promoting retinal reattachment rather than in enhancing short-term visual acuity. At 1 day post-operatively, both uncorrected visual acuity (UCVA) and best-corrected visual acuity (BCVA) in the control group showed significant improvement compared to pre-operative levels, with statistically significant differences. In contrast, no significant improvement was observed in the study group. This may be attributed to the differing refractive indices of C3F8 gas and aqueous humour. This refractive index disparity causes significant refraction and scattering of light as it passes through C3F8 gas and aqueous humour, thereby impairing light focusing and imaging. Consequently, the image formed on the retina becomes blurred (24). Furthermore, studies indicate that following C3F8 gas filling, the a-wave and b-wave components of the electroretinogram (ERG) exhibit a decline in the immediate post-operative period. This suggests functional suppression of retinal photoreceptor and bipolar cells, leading to transient alterations in retinal function that subsequently impact short-term visual acuity improvement (45). In the study group, patients' mean UCVA improved from (1.88 ± 0.37) logMAR to (0.60 ± 0.26) logMAR, and mean BCVA improved from (1.76 ± 0.54) logMAR to (0.55 ± 0.27) logMAR. These results suggest that over time, combined procedure can lead to increasingly significant improvements in patients' vision, thereby contributing to better visual function and overall quality of life.

The mean IOP of patients in both groups at 1 day, 1 week, 1 month, and 3 months after surgery remained within the normal range and was slightly higher than the pre-operative values. The increase in mean IOP was most marked on the first post-operative day, after which it gradually decreased and stabilised. In this study, seven patients developed elevated IOP on the first post-operative day. In the control group, acute IOP elevation was likely related to short-term ocular inflammation and swelling. In the study group, IOP elevation was attributed not only to inflammation and swelling but also to intraocular volume increase from gas expansion and transient angle closure caused by forward displacement of the iris–lens diaphragm (24). In all cases, IOP normalised after administration of topical and systemic hypotensive medication. No other complications such as retinal incarceration, haemorrhage, anterior segment ischaemia, or infection occurred during follow-up.

Therefore, the combination of SB and C3F8 is clinically valuable in the management of CRD. SB provides external indentation on the retina, closing retinal breaks and reducing vitreoretinal traction, while C3F8 filling delivers continuous internal tamponade to promote retinal reattachment and apposition. Used together, these approaches complement one another, enhancing both external and internal support, thereby increasing the success rate of retinal reattachment and improving patients' long-term prognosis.

Although this study demonstrated favourable outcomes with SB combined with C3F8 in the treatment of CRD, several limitations should be acknowledged. This retrospective cohort study cannot entirely preclude the influence of confounding bias. Although we employed methods such as propensity score matching and multivariate regression analysis to control for known confounding factors, the possibility of residual confounding remains unavoidable. Further rigorous prospective cohort studies or randomised controlled trials will be required in the future to validate the conclusions of this research. While clinical data were collected and analysed in detail, uncontrollable factors such as inter-individual variability may have influenced the results, thereby limiting the generalisability and reliability of the conclusions. The study sample size was relatively small, with only 40 patients included, which may not fully represent the broader patient population and could reduce the ability to detect rare complications or less common clinical scenarios.

Furthermore, the follow-up period was relatively short. Longer-term studies are needed to evaluate the sustained effects of SB combined with C3F8, particularly with respect to the stability of visual acuity, durability of retinal reattachment, and the incidence of late complications over 5–10 years or more. Future research should therefore incorporate larger cohorts and extended follow-up to provide more robust evidence regarding the long-term safety and effectiveness of this combined surgical approach.

SB combined with C3F8 for CRD appears to be a safe and effective treatment, producing significant improvements in visual acuity, a higher rate of retinal reattachment, lower recurrence rates, and a reduced risk of complications.

## Data Availability

The raw data supporting the conclusions of this article will be made available by the authors, without undue reservation.

## References

[1] Feltgen N Walter P. Rhegmatogenous retinal detachment–an ophthalmologic emergency. Dtsch Arztebl Int. (2014) 111:12–21. doi: 10.3238/arztebl.2014.0012 24565273 PMC3948016

[2] Xiong J Tran T Waldstein SM Fung AT. A review of rhegmatogenous retinal detachment: past, present and future. Wien Med Wochenschr. (2025) 175:186–202. doi: 10.1007/s10354-025-01085-9 40183886 PMC12031774

[3] Ghasemi Falavarjani K Alemzadeh SA Modarres M Alemzadeh SA Parvarash MM Naseripour M . Outcome of surgery in patients with giant retinal tear: 10 years experience. Eye. (2017) 31:1284–9. doi: 10.1038/eye.2017.145 28776588 PMC5601441

[4] Yokoyama S Kojima T Mori T Matsuda T Sato H Yoshida N . Clinical outcomes of endoscope-assisted vitrectomy for treatment of rhegmatogenous retinal detachment. Clin Ophthalmol. (2017) 11:2003–10. doi: 10.2147/OPTH.S147690 29180845 PMC5694206

[5] Mohamed YH Ono K Kinoshita H Uematsu M Tsuiki E Fujikawa A . Success rates of vitrectomy in treatment of rhegmatogenous retinal detachment. J Ophthalmol. (2016) 2016:2193518. doi: 10.1155/2016/2193518 27478632 PMC4961815

[6] Mete M Maggio E Ramanzini F Guerriero M Airaghi G Pertile G. Microstructural macular changes after pars plana vitrectomy for primary rhegmatogenous retinal detachment. Ophthalmologica. (2021) 244:551–9. doi: 10.1159/000517880 34167115

[7] Guner ME Guner MK Cebeci Z Kir N. Preoperative and postoperative factors affecting functional success in anatomically successful retinal detachment surgery. Korean J Ophthalmol. (2022) 36:477–85. doi: 10.3341/kjo.2022.0057 36220639 PMC9745350

[8] Sultan ZN Agorogiannis EI Iannetta D Steel D Sandinha T. Rhegmatogenous retinal detachment: a review of current practice in diagnosis and management. BMJ Open Ophthalmol. (2020) 5:e000474. doi: 10.1136/bmjophth-2020-000474 33083551 PMC7549457

[9] Nemet A Moshiri A Yiu G Loewenstein A Moisseiev E. A review of innovations in rhegmatogenous retinal detachment surgical techniques. J Ophthalmol. (2017) 2017:4310643. doi: 10.1155/2017/4310643 28584664 PMC5444028

[10] Governatori L Scampoli A Culiersi C Bernardinelli P Picardi SM Sarati F . Chandelier-assisted scleral buckling: a literature review. Vision. (2023) 7:47. doi: 10.3390/vision7030047 37489326 PMC10366817

[11] Rodriguez FJ Lewis H Kreiger AE Yoshizumi MO Sidikaro Y. Scleral buckling for rhegmatogenous retinal detachment associated with severe myopia. Am J Ophthalmol. (1991) 111:595–600. doi: 10.1016/S0002-9394(14)73705-5 2021169

[12] Shanmugam PM Ramanjulu R Mishra KCD Sagar P. Novel techniques in scleral buckling. Indian J Ophthalmol. (2018) 66:909–15. doi: 10.4103/ijo.IJO_136_18 29941729 PMC6032754

[13] Park SW Lee JJ Lee JE. Scleral buckling in the management of rhegmatogenous retinal detachment: patient selection and perspectives. Clin Ophthalmol. (2018) 12:1605–15. doi: 10.2147/OPTH.S153717 30214145 PMC6124476

[14] Taheri N Mousavi F Ahoor MH Latifi A Hedayati F. Management of uncomplicated primary retinal rhegmatogenous detachment. Int Ophthalmol. (2021) 41:1709–16. doi: 10.1007/s10792-021-01729-w 33511514

[15] Fan FF Xiao C Wang L Wang XT Liu DD Ge X . Efficacy of scleral buckling for the treatment of rhegmatogenous retinal detachment using a novel foldable capsular buckle. Int J Ophthalmol. (2024) 17:558–63. doi: 10.18240/ijo.2024.03.19 38721507 PMC11074169

[16] Lindsell LB Sisk RA Miller DM Foster RE Petersen MR Riemann CD . Comparison of outcomes: scleral buckling and pars plana vitrectomy versus vitrectomy alone for primary repair of rhegmatogenous retinal detachment. Clin Ophthalmol. (2017) 11:47–54. doi: 10.2147/OPTH.S112190 28053500 PMC5189967

[17] Cruz-Pimentel M Huang CY Wu L. Scleral buckling: a look at the past, present and future in view of recent findings on the importance of photoreceptor re-alignment following retinal re-attachment. Clin Ophthalmol. (2022) 16:1971–84. doi: 10.2147/OPTH.S359309 35733617 PMC9208732

[18] Jung YH Park KH Woo SJ Joo K Kim MS. Scleral buckling with adjuvant pneumatic retinopexy versus scleral buckling alone for rhegmatogenous retinal detachment. Sci Rep. (2024) 14:5249. doi: 10.1038/s41598-024-55999-2 38438557 PMC10912704

[19] Issa R Xia T Zarbin MA Bhagat N. Silicone oil removal: post-operative complications. Eye. (2020) 34:537–43. doi: 10.1038/s41433-019-0551-7 31406357 PMC7042253

[20] Cicinelli MV Benatti E Starace V Nadin F Di Nisi E Bandello F . Recurrences and macular complications after perfluorocarbon-liquid-free vitrectomy for primary rhegmatogenous retinal detachment. Ophthalmol Ther. (2023) 12:3219–32. doi: 10.1007/s40123-023-00811-z 37775683 PMC10640444

[21] Hilton GF Grizzard WS. Pneumatic retinopexy A two-step outpatient operation without conjunctival incision. Ophthalmology. (1986) 93:626–41. doi: 10.1016/S0161-6420(86)33696-0 3523357

[22] Kunikata H Abe T Nakazawa T. Historical, current and future approaches to surgery for rhegmatogenous retinal detachment Tohoku. J Exp Med. (2019) 248:159–68. doi: 10.1620/tjem.248.159 31308289

[23] Chan CK Lin SG Nuthi AS and Salib DM. Pneumatic retinopexy for the repair of retinal detachments: a comprehensive review (1986-2007). Surv Ophthalmol. (2008) 53:443–78. doi: 10.1016/j.survophthal.2008.06.008 18929759

[24] Kanclerz P Grzybowski A. Complications associated with the use of expandable gases in vitrectomy. J Ophthalmol. (2018) 2018:8606494. doi: 10.1155/2018/8606494 30581605 PMC6276446

[25] Shettigar MP Dave VP Chou HD Fung A Iguban E March de. Ribot F, et al. Vitreous substitutes and tamponades - a review of types, applications, and future directions. Indian J Ophthalmol. (2024) 72:1102–11. doi: 10.4103/IJO.IJO_2417_23 39078953 PMC11451774

[26] Zhang X Wu H Guo S Zhu T Xiang Z Li G . Modification of the pneumatic retinopexy for the treatment of rhegmatogenous retinal detachment with multiple-quadrant retinal breaks. Front Med. (2025) 12:1549152. doi: 10.3389/fmed.2025.1549152 40144872 PMC11937036

[27] Kontos A Tee J Stuart A Shalchi Z Williamson TH. Duration of intraocular gases following vitreoretinal surgery. Graefes Arch Clin Exp Ophthalmol. (2017) 255:231–6. doi: 10.1007/s00417-016-3438-3 27460279

[28] Pinto A Soares DTC Costa MR Lira RPC. Influence of the dilution method on the intraocular duration of C3F8 in vitrectomy for macular hole: a randomized clinical trial. Arq Bras Oftalmol. (2024) 87:e20220336. doi: 10.5935/0004-2749.2022-0336 38451686 PMC11620541

[29] Wiley ZC Huang X Staggers KA Hamill MB. Comparison of 20% SF6 and 6% C3F8 gas for anterior chamber tamponade in endothelial keratoplasty. Cornea. (2024) 43:1238–44. doi: 10.1097/ICO.0000000000003469 38251881 PMC11365599

[30] Tan S Wu S Miao J Han J Jin W Li D . A short-time real-world study of two perfluoropropane tamponade methods in pars plana vitrectomy for retinal detachment. Ophthalmic Res. (2023) 66:1300–7. doi: 10.1159/000534208 37812921 PMC10627491

[31] Saleh OA Fleissig E Barr CC. Outcomes after the use of silicone oil in complex retinal detachment repair. J Vitreoretin Dis. (2020) 4:96–102. doi: 10.1177/2474126419896658 37008378 PMC9976256

[32] Pablo L Garay-Aramburu G Garcia Layana A Fernandez A Vazquez I Acebes X . Assessing the economic burden of vision loss and irreversible legal blindness in Spain (2021-2030): a societal perspective. Health Econ Rev. (2024) 14:70. doi: 10.1186/s13561-024-00546-y 39225974 PMC11370269

[33] Mozaffarieh M Sacu S Benesch T Wedrich A. Mental health measures of anxiety and depression in patients with retinal detachment. Clin Pract Epidemiol Ment Health. (2007) 3:10. doi: 10.1186/1745-0179-3-10 17640389 PMC2031884

[34] Chaudhary R Scott RAH Wallace G Berry M Logan A Blanch RJ. Inflammatory and fibrogenic factors in proliferative vitreoretinopathy development. Transl Vis Sci Technol. (2020) 9:23. doi: 10.1167/tvst.9.3.23 32742753 PMC7357815

[35] Moysidis SN Thanos A Vavvas DG. Mechanisms of inflammation in proliferative vitreoretinopathy: from bench to bedside. Mediators Inflamm. (2012) 2012:815937. doi: 10.1155/2012/815937 23049173 PMC3463807

[36] Popovic MM Muni RH Nichani P Kertes PJ. Pars plana vitrectomy, scleral buckle, and pneumatic retinopexy for the management of rhegmatogenous retinal detachment: a meta-analysis. Surv Ophthalmol. (2022) 67:184–96. doi: 10.1016/j.survophthal.2021.05.008 34043984

[37] Kasetty VM Monsalve PF Sethi D Yousif C Hessburg T Kumar N . Cataract progression after primary pars plana vitrectomy for uncomplicated rhegmatogenous retinal detachments in young adults. Int J Retina Vitreous. (2024) 10:19. doi: 10.1186/s40942-024-00538-4 38383511 PMC10882894

[38] Dou Z Han J Zhao S. Efficacy of a simple intravitreal perfluoropropane injection in treating unclosed idiopathic macular holes following vitrectomy. BMC Ophthalmol. (2025) 25:61. doi: 10.1186/s12886-024-03839-2 39910548 PMC11800614

[39] Choi M Hong S Yun C Kim SW. Objective analysis of perfluoropropane tamponade area after pars plana vitrectomy using ultra-widefield fundus stereographic projection images. Sci Rep. (2020) 10:18268. doi: 10.1038/s41598-020-75493-9 33106589 PMC7588468

[40] Abdelkader AME Abouelkheir HY. Supine positioning after vitrectomy for rhegmatogenous retinal detachments with inferior retinal breaks. Int J Retina Vitreous. (2020) 6:41. doi: 10.1186/s40942-020-00247-8 32944286 PMC7490905

[41] Ghoraba HH Leila M Shebl M Abdelhafez MA Abdelfattah HM. Long-term outcome after silicone oil removal in eyes with myopic retinal detachment associated with macular hole. Clin Ophthalmol. (2021) 15:1003–11. doi: 10.2147/OPTH.S298565 33727783 PMC7953888

[42] Schoneberger V Li JQ Menghesha L Holz FG Schaub F Krohne TU. Outcomes of short- versus long-acting gas tamponades in vitrectomy for rhegmatogenous retinal detachment. Int J Retina Vitreous. (2024) 10:16. doi: 10.1186/s40942-024-00530-y 38317211 PMC10840190

[43] Agrawal S Shanmugam PM Mishra DK Ramanjulu R Ravishankar HN Sagar P . Volume measurement of the vitrectomised eye and its applications in practice. Indian J Ophthalmol. (2024) 72:1501–5. doi: 10.4103/IJO.IJO_3237_23 39331442 PMC11573019

[44] Peck TJ Starr MR Yonekawa Y Khan MA Obeid A Ryan EH . Outcomes of primary rhegmatogenous retinal detachment repair in eyes with preoperative grade B or C proliferative vitreoretinopathy. J Vitreoretin Dis. (2022) 6:194–200. doi: 10.1177/24741264211046770 37008548 PMC9976120

[45] Wang J Li Q. Experiment of vitreous liquefaction induced by C3F8. Pak J Pharm Sci. (2017) 30:281–7. 28625955

